# Epigenetic Regulation of Megaspore Mother Cell Formation

**DOI:** 10.3389/fpls.2021.826871

**Published:** 2022-02-03

**Authors:** Ting Jiang, Binglian Zheng

**Affiliations:** State Key Laboratory of Genetic Engineering, Ministry of Education Key Laboratory of Biodiversity Sciences and Ecological Engineering, Collaborative Innovation Center of Genetics and Development, School of Life Sciences, Fudan University, Shanghai, China

**Keywords:** MMC, epigenetic regulation, small RNA, DNA methylation, ovule development epigenetic regulation in MMC

## Abstract

In flowering plants, the female gametophyte (FG) initiates from the formation of the megaspore mother cell (MMC). Among a pool of the somatic cells in the ovule primordium, only one hypodermal cell undergoes a transition of cell fate to become the MMC. Subsequently, the MMC undergoes a series of meiosis and mitosis to form the mature FG harboring seven cells with eight nuclei. Although *SPL/NZZ*, the core transcription factor for MMC formation, was identified several decades ago, which and why only one somatic cell is chosen as the MMC have long remained mysterious. A growing body of evidence reveal that MMC formation is associated with epigenetic regulation at multiple layers, including dynamic distribution of histone variants and histone modifications, small RNAs, and DNA methylation. In this review, we summarize the progress of epigenetic regulation in the MMC formation, emphasizing the roles of chromosome condensation, histone variants, histone methylation, small RNAs, and DNA methylation.

## Introduction

Different from that in animals, the germline cells are not specialized during embryo development in plants. Instead, when plants grow from vegetative growth to reproductive growth, several specific somatic cells undergo cell fate transition to become the germline cells. In flowering plants, the male and female gametophytes (FGs) develop within the anther and the ovule, respectively. In most angiosperms and gymnosperms, only one somatic cell in the nucellus region of the ovule changes its cell identity and later becomes the megaspore mother cell (MMC). MMC undergoes two meiotic divisions to give rise to four megaspores. Then, only one megaspore near the chalaza becomes the functional megaspore (FM), while the other three cells undergo programmed cell death. Subsequently, the FM undergoes three mitotic nuclear divisions, finally resulting in the formation of a mature FG, so called embryo sac (Grossniklaus and Schneitz, 1998). As the first step of FG development, cell fate transition of MMC is of great importance.

**FIGURE 1:**
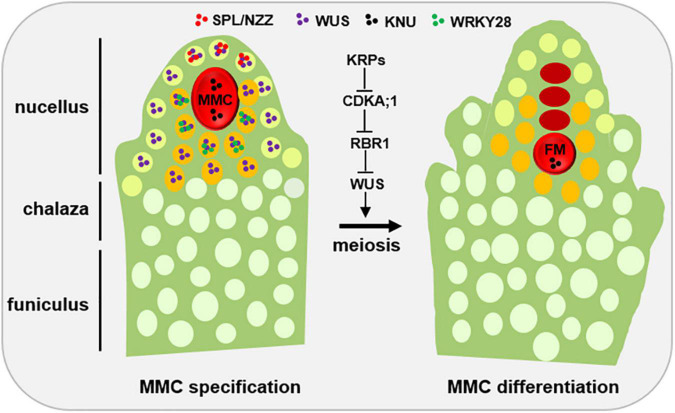
Developmental schematic of MMC formation. In the early ovule (left), several pioneer transcription factors, mainly SPL/NZZ and WUS, play an important role in promoting MMC formation. The spatial distribution of SPL, WUS and WRKY28 away from the centered position of the nucellus region is the prerequisite of MMC specification and MMC differentiation, respectively. KNU, a widely used MMC marker. Once the MMC specification is finished, the KRP-CDKA;1-RBR1 pathway plays a key role to ensure the MMC into meiotic rather than mitotic competency by inhibiting the expansion of WUS into the MMC. L1 cell, light green; L2 cell, orange; MMC, megaspore mother cell, red; FM, functional megaspore, red. Other distal somatic cells are indicated in light white.

In Arabidopsis, the pre-meiosis ovule can be divided into three parts along a proximal–distal axis, including nucellus, chalaza, and funiculus (Schneitz et al., 1997). The cells in the nucellus region can be further divided into two layers, the epidermal layer (L1) and the subepidermal layer (L2). In general, the archespore that arises from the most distal cell in L2 changes its cell fate to develop into MMC. Subsequently, the MMC becomes recognizable as a single, large, and elongated subepidermal cell, which is centrally positioned within the nucellus and displays a prominent nucleus and nucleolus (Schneitz et al., 1997; Hernandez-Lagana et al., 2021; Figure 1). However, the mechanism of MMC formation remains unclear, especially, which, why, and how only one somatic cell is allowed to become MMC? In general, MMC formation is thought to be controlled by two steps: first, restricting only one cell differentiation to MMC, and second, preventing self-renewal of the designated MMC before meiosis. Here, we review major advances in the cell fate control of MMC, emphasizing the roles of epigenetic regulations, including the change of chromosome condensation status, distribution of histone variants and histone modifications, small RNA biogenesis, and DNA methylation.

## Key Developmental Regulators of Megaspore Mother Cell Formation

*SPOROCYTELESS/NOZZLE* (*SPL/NZZ*), a MADS-box transcriptional factor, is the first gene which was found to play a pioneer role in MMC formation, as the *spl/nzz* mutants have smaller nucellus and the archespore completely fails to undergo differentiation resulting in the complete absence of the MMC (Schiefthaler et al., 1999; Yang et al., 1999; Balasubramanian and Schneitz, 2000). In contrast, a recent study shows that ectopic expression of *SPL/NZZ* caused additional enlarged MMC-like cells in the early ovules (Mendes et al., 2020). Of note, as a pioneer transcription factor in germline formation, SPL/NZZ is also required for male gametophyte development, as microsporocyte formation was blocked in the *SPL/NZZ* mutants (Schiefthaler et al., 1999; Yang et al., 1999). The homologs of *SPL/NZZ* in tomato and rice are also essential for both male and FG development (Rojas-Gracia et al., 2017; Ren et al., 2018). SPL/NZZ uses its EAR motif to recruit co-repressor TOPLESS, to regulate sporocyte formation (Chen et al., 2014; Wei et al., 2015). Moreover, WUSCHEL (WUS), a key regulator for stem cell fate in plants, acts in concert with SPL/NZZ to contribute MMC formation (Lieber et al., 2011). Based on the observations that *SPL/NZZ* is mainly expressed at the tip of the ovule primordium (Mendes et al., 2020; Zhao et al., 2020), and WUS preferentially accumulates in the nucellar cells surrounding the MMC (Zhao et al., 2017; He et al., 2019; Mendes et al., 2020), it is thought that the roles of both SPL/NZZ and WUS in regulating MMC formation are non-cell-autonomous (Figure 1).

Once MMC specification is determined, the MMC undergoes meiosis to produce the four megaspores and only one of megaspores called FM develops into the mature FG via several rounds of mitoses (Grossniklaus and Schneitz, 1998). However, why the MMC is able to switch mitotic division to meiotic division? Cyclin-dependent kinase (CDK) inhibitor KIP-RELATED PROTEIN (KRP) family inhibit CDKA;1 to ensure the entry of MMC into meiosis rather than mitosis (Zhao et al., 2017; Figure 1). By analyzing MMC formation in the triple mutant of *KRP*, Zhao et al. (2017), shows that KRPs are essential for the restriction of the plant germline harboring only one MMC per ovule by inhibiting CDKA;1. Furthermore, CDKA;1 targets *RETINOBLASTOMA-RELATED 1* (*RBR1*), a Retinoblastoma (Rb) homolog in Arabidopsis (Ebel et al., 2004), to inhibit the designated meiocytes entering mitosis (Zhao et al., 2017). As a result, the meiocytes of the *rbr1* mutants undergo several mitotic divisions, resulting in the formation of supernumerary meiocytes that give rise to multiple MMCs per ovule (Zhao et al., 2017). Intriguingly, the expression of *WUS* expands from the surrounding somatic cells to the MMC in both *krp* and *rbr1* mutants (Zhao et al., 2017). Moreover, loss-of-function of *WUS* significantly restored the phenotype of multiple MMCs in the *rbr1* mutants (Zhao et al., 2017). However, ectopic expression of *WUS* failed to induce the entry of MMC into mitotic divisions, suggesting that RBR1 not WUS is a central hub to determine the switch of MMC differentiation (Zhao et al., 2017). In addition, RBR1 represses cell cycle regulator E2F transcription factors to regulate the cell fate of MMC, as the *e2f* mutant harbors two to three MMCs per ovule primordium (Yao et al., 2018). Altogether, these findings indicate that not only MMC specification but also MMC differentiation are tightly regulated (Figure 1).

## De-Condensed Chromatin and Decreased Heterochromatin in the Megaspore Mother Cell

Once a specific somatic cell is chosen to develop into the MMC, both the cell itself, the nucleus, and even the nucleolar of the MMC increase significantly in size (Schneitz et al., 1997), which mark the MMC distinguishable clearly from the surrounding somatic cells. Chromatin condensation and heterochromatin formation are usually correlated to the nucleus size (van Zanten et al., 2011; Wang et al., 2013). Using non-denaturing whole-mount DNA staining and confocal imaging, She et al. (2013) showed the MMC exhibits a 60% reduction in heterochromatin content and a decreased number of chromocenters, indicating that a quick establishment of a MMC-specific chromatin state.

**FIGURE 2:**
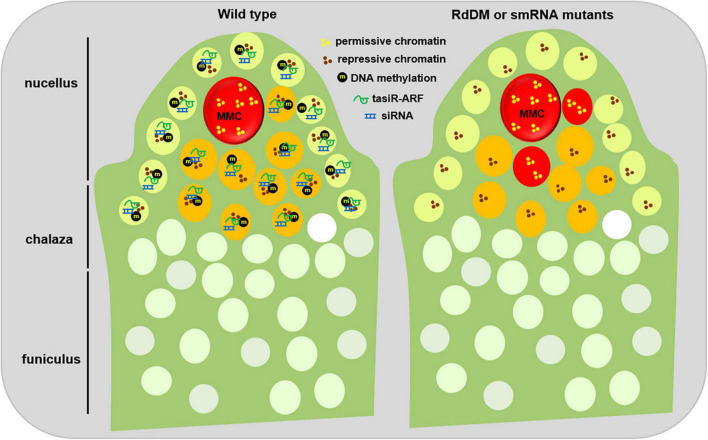
Epigenetic regulation of MMC formation. During early ovule development of the wild type plants (left), small RNA, mainly tasiRNA and siRNA, are widely produced. On one hand, tasiRNAs impede the surrounding somatic cells to activate their potential germline identity by inhibiting ARF3 to the nucellus region; on the other hand, siRNAs reinforce the repressive chromatin status of the surrounding cells by guarding DNA methylation and/or histone modification. Meanwhile, several inactive histone modifications, such as H1, H3K9me2, and H3K27me3, facilitate the maintenance of repressive chromatin status in the surrounding cells. In contrast, the MMC shows a higher level of active histone modifications, for example, H3K4me3, thus keeping a permissive chromatin state. When the activities of small RNAs or DNA methylation are disrupted (right), the surrounding somatic cells adjacent to the MMC obtain a permissive chromatin state, and switch to the germline cell identity. This epigenetic dimorphism between the surrounding somatic cells and the MMC might ensure the establishment of a permission chromatin status in the MMC. L1 cell, light green; L2 cell, orange; MMC, megaspore mother cell, red.

Histone H1, a linker histone, establishes the compaction state of an array of nucleosomes to influence the status of chromatin condensation (Osipova et al., 1980). In Arabidopsis, H1 is encoded by three genes, *H1.1*, *H1.2*, and *H1.3* (Ascenzi and Gantt, 1997). *H1.1* and *H1.2* are significantly down-regulated in the MMC, and *H1.3* is barely detected in the ovule primordia (She et al., 2013). Moreover, H1.1 and H1.2 are *de novo* incorporated into the chromatin for condensation as meiosis occurs (She et al., 2013), suggesting that the decrease of H1 might be the consequence after a somatic cell is specialized into the MMC (Figure 2).

Consistent with the role of H1 in chromosome condensation, loss-of-function of *H1* causes a global decrease of heterochromatin formation and transposon silencing (Zemach et al., 2013; He et al., 2019). In plants, heterochromatin formation is usually associated with decreased active histone modifications, for example, H3K4me3, and increased inactive histone modifications, such as H3K27me3 and H3K9me2 (Bender, 2004). Immunofluorescence assays show, in contrast to those in the surrounding cells, H3K4me3 is enriched to 2.7-fold in the MMC while H3K27me1, H3K9me2, and H3K27me3 reduced in the MMC (She et al., 2013), indicating a permissive chromatin environment of the MMC (Figure 2). Correspondingly, SET DOMAIN GROUP 2 (SDG2), a writer for H3K4me3 (Berr et al., 2010; Guo et al., 2010), and LHP1, a key regulator for H3K27me3, are highly and barely expressed in the MMC, respectively (She et al., 2013). These observations indicate that with the increase of both cell size and nuclear even nucleolar size, histone modifications are actively regulated to establish a unique permission chromatin environment for the MMC.

## Histone Variants Are Actively Exchanged to Concert the Megaspore Mother Cell Chromatin Status

Accompanied with chromatin condensation, histone variants often confer specific structure and functional chromatin features due to their substitutable capacity for the core canonical histone in nucleosomes in eukaryotes. Among multiple histone variants, histone H3 is encoded by *HISTONE THREE RELATED (HTR)* gene family containing 15 members in *Arabidopsis* (Okada et al., 2005). *HTR12*, a centromere-specific H3 variant CENH3 (Talbert et al., 2002), was ubiquitously expressed in the MMC (Ravi et al., 2011; She et al., 2013). By contrast, HTR8 and HTR5, two H3.3 variants that are usually associated with transcriptional competence (Ingouff et al., 2010), are specifically expressed in the MMC (She et al., 2013). HTR13, a H3.1 variant that is usually related inactive transcription activity (Jacob et al., 2014), can be gradually evicted in multiple L2 cells of the nucellus during early ovule development, but this eviction was only limited to the MMC once the identity of the MMC is designated (Hernandez-Lagana and Autran, 2020; Figure 2). The eviction of H3.1 in the MMC indicates that H3.1 can act as a marker to distinguish cell identity, which also happens in the root quiescent center (Otero et al., 2016). The phenomenon of multiple early L2 cells with H3.1 eviction suggests that not only one L2 cell has acquired the potential to turn into the germline cell, but finally only one can switch to the MMC by an unknown mechanism (Hernandez-Lagana and Autran, 2020).

In contrast to H3 variants, HTA11, a H2A.Z variant, is evicted from the early MMC but reincorporated later (She et al., 2013). Moreover, *WRKY28*, a transcription factor labeling the L2 cells, is activated by cytochrome P450 gene *KLU* through the chromatin remodeling complex SWR1-mediated H2A.Z deposition (Qin et al., 2014; Zhao et al., 2018). Therefore, although the mechanism by which specific chromatin hallmarks are differentially regulated in the MMC is unknown, the highly dynamic exchange among H1, H3.1, H3.3, H2A.Z, and CENH3 is consistent with a global pattern of chromatin de-condensation in the MMC, indicating that a specific chromatin reprogramming during MMC specification and differentiation (Figure 2).

## Small RNA Negatively Regulates Megaspore Mother Cell Formation

Based on the modes of biogenesis and action, small RNAs in plants are usually divided into three groups: microRNA (miRNA), small interfering RNA (siRNA), and trans-acting siRNA (tasiRNA) (Borges and Martienssen, 2015). In general, *MIRNA* genes are transcribed into hairpin structured-precursor RNAs followed by Dicer-like 1 (DCL1)-mediated twice cleavages to produce 21–24 nt miRNAs, then miRNAs are mainly loaded onto Argonaute 1 (AGO1) for target gene inhibition with sequence complementarity (Rogers and Chen, 2013). siRNAs are mainly originated from heterochromatic regions, including transposable elements (TE) and DNA repeats. The heterochromatic regions are transcribed into double-stranded RNA precursors by Pol II-RDR6 (RNA-Dependent RNA Polymerase 6) or Pol IV-RDR2. Then, these precursors are cleaved by DCL3 to produce 21–24 nt siRNAs, which are mainly loaded onto AGO4 with the guidance of Pol V-transcribed scaffold RNAs. Lastly, the AGO4-siRNA complex recruits *de novo* DNA methyltransferase DRM2 to initiate DNA methylation for heterochromatic silencing (Matzke and Mosher, 2014). The siRNA pathway is called RdDM (RNA-directed DNA methylation). In contrast to miRNA and siRNA, tasiRNA biogenesis is initiated from specific miRNA-mediated target cleavage processes, in which non-coding *TAS* transcripts are cleaved by AGO1-miR173 or AGO7-miR390, then the cleavage products are copied into double-stranded RNAs by RDR6 with the help of SGS3 (Suppressor of Gene Silencing 3), finally these double-stranded RNAs are diced into 21 or 24 nt tasiRNA by DCL4 (Allen et al., 2005). Similar to miRNA, tasiRNAs are mainly loaded onto AGO1 to inhibit target genes.

By focusing on the function of those genes highly expressed in the FG, AGO9 was first isolated due to additional enlarged MMC-like cell formation in the *ago9* mutants (Olmedo-Monfil et al., 2010). Subsequently, further genetic analysis show that AGO4, AGO6, AGO8, other three components of the same subclass of AGO9, are all involved in MMC formation (Hernandez-Lagana et al., 2016). Consistent with the function of AGO9 in the siRNA pathway, Pol IV, RDR2, and DCL3, three key genes responsible for siRNA biogenesis, all exhibited increased incidence of additional MMC-like cells (Olmedo-Monfil et al., 2010). Of note, different ecotypes of Arabidopsis exhibit differences in the numbers of MMC, and this variation is largely correlated to the pattern differences of transcriptional regulation and localization of AGO9 in the MMC among ecotypes (Rodriguez-Leal et al., 2015). These observations demonstrate that the siRNA pathway is required to restrict the differentiation of sub-epidermal cells into the MMC in pre-meiotic ovules.

Besides those mutants in the siRNA pathway, the *rdr6*, *mir390*, *ago7*, *tas3*, mutants that affect tasiRNA biogenesis, also exhibits additional MMC-like cells per ovule (Olmedo-Monfil et al., 2010; Su et al., 2020). By screening new genes acting with RDR6 together to restrict MMC formation, TEX1, HPR1, and THOC6, several components of the THO/TREX complex, were identified as their corresponding mutants exhibit additional MMC-like cells in some pre-meiosis ovules (Su et al., 2017). The isolation of the *tho/trex* mutants is not surprising because the THO/TREX complex, similar to RDR6, is required for tasiRNA biogenesis (Jauvion et al., 2010; Yelina et al., 2010). Further evidence shows that tasiRNA inhibits the surrounding L2 cells into the MMC by restricting the expression of *Auxin Responsive Factor 3 (ARF3)* to the nucellus region (Su et al., 2017, 2020). Ectopic expression of ARF3 with *TAS3* binding site mutation in the lateral epidermal cells caused multiple MMC cells per ovule primordium (Su et al., 2020), suggesting that the inhibition of ARF3 is prerequisite for the restriction of one MMC per primordium. Moreover, these enlarged MMC-like cells of the tasiRNA mutants showed expression of *KNU*, a marker gene for MMC (Payne et al., 2004), indicating that these additional enlarged MMC-like cells have acquired the identity of MMC (Su et al., 2017). Collectively, these findings uncover the role of two small-RNA pathways in the restriction of MMC specification and differentiation (Figure 2).

## DNA Methylation Negatively Regulates Megaspore Mother Cell Formation

Since the siRNA pathway is required for MMC formation, and siRNA plays a role in gene silencing *via* guiding DNA methylation in plants, i.e., RdDM (Matzke and Mosher, 2014). However, little is known about DNA methylation dynamics during reproduction largely due to the technical difficulty of isolating pure and sufficient germ cells for evaluation. By developing two live imaging sensors targeting CG (MBD-Venus) and non-CG (SUVH9-Venus) methylation, respectively, Ingouff et al. (2017) showed that in contrast to the relative steady level of CG methylation during whole MMC formation, CHH methylation became undetectable in the MMC. The reduced levels of DNA methylation correlate with the de-condensed chromatin status and reduced heterochromatin formation in the MMC.

Besides siRNA biogenesis machinery (Pol IV, RDR2, and DCL3) and siRNA effectors AGO4, AGO6, and AGO9 have been involved in MMC formation, a recent finding show that the *de novo* DNA methyltransferases DRM1 and DRM2 are required for the restriction of additional MMC formation (Mendes et al., 2020), further indicating that the RdDM pathway is necessary for MMC specification and differentiation. Interestingly, *SEEDSTICK (STK)*, a MADS-box transcription factor controlling the ovule identity, binds to the CArG-box regions of *AGO9* and *RDR6* to promote their expression, and finally promoting expression of *SPL/NZZ* (Mendes et al., 2020). Moreover, in contrast to that the expression of *SPL/NZZ* is confined to the tip of early ovule/L1 layer in the wild type plants, *SPL/NZZ* ectopically expands throughout the distal nucellar primordium in the *ago9* and *drm1drm2* mutants (Mendes et al., 2020). The establishment of the STK-RdDM-SPL/NZZ relay provides direct evidence how RdDM activities is integrated by both upstream and downstream transcription factors during a specific developmental process.

Although *MET1*, a DNA methyltransferase responsible for CG methylation in Arabidopsis (Xiao et al., 2003), is ubiquitously expressed during MMC formation (Ingouff et al., 2017; Li et al., 2017), the *met1* mutant exhibits additional MMC-like cells per ovule (Li et al., 2017). Moreover, ARID1 (ARID domain-containing 1), a transcription factor that is required for heterochromatic silencing and sperm cell formation (Zheng et al., 2014), regulates MET1 reciprocally in the gamete cells, and also inhibit MMC formation (Li et al., 2017). In addition, ARID1 acts with AGO9 together to mediate siRNA movement in male gametes (Wu et al., 2021). The fact that multiple heterochromatin regulators, for example, RdDM factors, H1, MET1, and ARID1, even *TRAF Mediated Gametogenesis Progression (TRAMGaP)*, an AGO9-interacting protein (Singh et al., 2017), negatively regulate MMC specification and differentiation, indicates that heterochromatin silencing restricts the potential germline identity of the surrounding somatic cells.

## Epigenetic Regulation of Megaspore Mother Cell Formation in Other Plants

Although most angiosperms and gymnosperms harbor only one MMC, some plant species naturally develop more than one MMC. For example, *Trimenia moorei*, an ancient angiosperm, exhibits multiple MMCs (Bachelier and Friedman, 2011). *Gnetum*, an atypical gymnosperm, forms up to 12 MMCs and 5 of them are able to even enter meiosis (Lora et al., 2019). Why these plants develop multiple MMCs? A recent finding shows that *Utricularia gibba*, a carnivorous plant, has an unusual distribution of small RNAs and reduced global DNA methylation levels (Cervantes-Perez et al., 2021). Intriguingly, a truncated DCL3 correlates with reduced small RNA levels and DNA methylation levels, and female gametogenesis abnormalities in *U. gibba* (Cervantes-Perez et al., 2021). This finding further provides evidence that small RNA activity might be a driving force for MMC specification. Moreover, *U. gibba* might be an ideal system to investigate the evolution relationship between the RdDM pathway and MMC numbers.

A previous study ever documented the effects of natural variation of epigenetic regulators on MMC development in different ecotype of Arabidopsis (Rodriguez-Leal et al., 2015). By comparing the frequency of multiple MMCs incidence F1 hybrids of specific ecotypes, the authors show that the transcriptional patterns and protein subcellular localization of AGO9 contribute to varied MMC development among different ecotypes to an extent (Rodriguez-Leal et al., 2015). Besides Arabidopsis, several lines of evidence further show that the core genes in the small RNA pathway and DNA methylation are possibly required for MMC development. For example, loss-of-function of *dmt102* and *dmt103*, two DNA methyltransferases in maize, caused apomictic ovule development (Garcia-Aguilar et al., 2010). AGO104, a homolog of AGO9 in maize, is also expressed in the somatic cells surrounding the MMC, and AGO104 is required for inhibiting the transition of the germline cells to the somatic cell (Singh et al., 2011). In pineapple, many genes in the RdDM pathway are highly expressed in the MMC-stage ovule (Zhao et al., 2021). Collectively, the existence of small RNA and DNA methylation-mediated gene silencing in various plant species and the expression of the corresponding genes in the ovule primordium indicate epigenetic regulation is a widely mechanism during MMC development.

## Conclusion and Perspective

Considering the importance of MMC as the first female germline cell lineage in plants, to understand how this specific cell is specialized and differentiated is especially central for plant reproductive development. Classical genetic strategies have identified that several key developmental factors promote MMC specification and differentiation, such as SPL/NZZ, KRPs, RBR1, and WUS. Individual analyses of specific epigenetic regulators and epigenetic modifications show that many genes related to small RNA biogenesis and activity, DNA methylation, heterochromatin silencing, histone variants, and histone modifications, are required for MMC formation by restricting the germline identity of the surrounding somatic cells. Future work about the nature of the very beginning trigger sensed by these key factors will provide us a blueprint of the mechanism for cell fate control in plants.

Based on the differential patterns of DNA methylation, small RNA activities, and in the distribution of histone variants and histone modifications between the MMC and the surrounding somatic cells, an epigenetic dimorphism is established during MMC specification and differentiation. This dimorphism of epigenetic reprogramming might be such an above-mentioned possible trigger. Therefore, it would be very useful to create an accurate map of epigenetic dimorphism during MMC formation, if the technique difficulty of isolating high quality single cells from the early ovule primordium can be overcome in the future.

## Author Contributions

All authors listed have made a substantial, direct, and intellectual contribution to the work, and approved it for publication.

## Conflict of Interest

The authors declare that the research was conducted in the absence of any commercial or financial relationships that could be construed as a potential conflict of interest.

## Publisher’s Note

All claims expressed in this article are solely those of the authors and do not necessarily represent those of their affiliated organizations, or those of the publisher, the editors and the reviewers. Any product that may be evaluated in this article, or claim that may be made by its manufacturer, is not guaranteed or endorsed by the publisher.

## References

[B1] Allen E. Xie Z. Gustafson A. M. Carrington J. C. (2005). microRNA-directed phasing during trans-acting siRNA biogenesis in plants. *Cell* 121 207–221. 10.1016/j.cell.2005.04.004 15851028

[B2] Ascenzi R. Gantt J. S. (1997). A drought-stress-inducible histone gene in *Arabidopsis thaliana* is a member of a distinct class of plant linker histone variants. *Plant Mol. Biol.* 34 629–641. 10.1023/a:1005886011722 9247544

[B3] Bachelier J. B. Friedman W. E. (2011). Female gamete competition in an ancient angiosperm lineage. *Proc. Natl. Acad. Sci. U.S.A.* 108 12360–12365. 10.1073/pnas.1104697108 21690400 PMC3145725

[B4] Balasubramanian S. Schneitz K. (2000). NOZZLE regulates proximal-distal pattern formation, cell proliferation and early sporogenesis during ovule development in *Arabidopsis thaliana*. *Development* 127 4227–4238. 10.1242/dev.127.19.4227 10976054

[B5] Bender J. (2004). Chromatin-based silencing mechanisms. *Curr .Opin. Plant Biol.* 7 521–526. 10.1016/j.pbi.2004.07.003 15337094

[B6] Berr A. McCallum E. J. Menard R. Meyer D. Fuchs J. Dong A. (2010). Arabidopsis SET DOMAIN GROUP2 is required for H3K4 trimethylation and is crucial for both sporophyte and gametophyte development. *Plant Cell* 22 3232–3248. 10.1105/tpc.110.079962 21037105 PMC2990135

[B7] Borges F. Martienssen R. A. (2015). The expanding world of small RNAs in plants. *Nat. Rev. Mol. Cell Biol.* 16 727–741. 10.1038/nrm4085 26530390 PMC4948178

[B8] Cervantes-Perez S. A. Yong-Villalobos L. Florez-Zapata N. M. V. Oropeza-Aburto A. Rico-Resendiz F. Amasende-Morales I. (2021). Atypical DNA methylation, sRNA-size distribution, and female gametogenesis in *Utricularia gibba*. *Sci. Rep.* 11:15725. 10.1038/s41598-021-95054-y 34344949 PMC8333044

[B9] Chen G. H. Sun J. Y. Liu M. Liu J. Yang W. C. (2014). SPOROCYTELESS is a novel embryophyte-specific transcription repressor that interacts with TPL and TCP proteins in *Arabidopsis*. *J. Genet. Genom.* 41 617–625. 10.1016/j.jgg.2014.08.009 25527103

[B10] Ebel C. Mariconti L. Gruissem W. (2004). Plant retinoblastoma homologues control nuclear proliferation in the female gametophyte. *Nature* 429 776–780. 10.1038/nature02637 15201912

[B11] Garcia-Aguilar M. Michaud C. Leblanc O. Grimanelli D. (2010). Inactivation of a DNA methylation pathway in maize reproductive organs results in apomixis-like phenotypes. *Plant Cell* 22 3249–3267. 10.1105/tpc.109.072181 21037104 PMC2990141

[B12] Grossniklaus U. Schneitz K. (1998). The molecular and genetic basis of ovule and megagametophyte development. *Semin. Cell Dev. Biol.* 9 227–238. 10.1006/scdb.1997.0214 9599420

[B13] Guo L. Yu Y. Law J. A. Zhang X. (2010). SET DOMAIN GROUP2 is the major histone H3 lysine 4 trimethyltransferase in *Arabidopsis*. *Proc. Natl. Acad. Sci. U.S.A.* 107 18557–18562. 10.1073/pnas.1010478107 20937886 PMC2972934

[B14] He S. Vickers M. Zhang J. Feng X. (2019). Natural depletion of histone H1 in sex cells causes DNA demethylation, heterochromatin decondensation and transposon activation. *Elife* 8:42530. 10.7554/eLife.42530 31135340 PMC6594752

[B15] Hernandez-Lagana E. Autran D. (2020). H3.1 eviction marks female germline precursors in *Arabidopsis*. *Plants* 9:322. 10.3390/plants9101322 33036297 PMC7600056

[B16] Hernandez-Lagana E. Mosca G. Mendocilla-Sato E. Pires N. Frey A. Giraldo-Fonseca A. (2021). Organ geometry channels reproductive cell fate in the *Arabidopsis* ovule primordium. *Elife* 10:66031. 10.7554/eLife.66031 33960300 PMC8219382

[B17] Hernandez-Lagana E. Rodriguez-Leal D. Lua J. Vielle-Calzada J. P. (2016). A multigenic network of ARGONAUTE4 clade members controls early megaspore formation in *Arabidopsis*. *Genetics* 204 1045–1056. 10.1534/genetics.116.188151 27591749 PMC5105840

[B18] Ingouff M. Rademacher S. Holec S. Soljic L. Xin N. Readshaw A. (2010). Zygotic resetting of the HISTONE 3 variant repertoire participates in epigenetic reprogramming in *Arabidopsis*. *Curr. Biol.* 20 2137–2143. 10.1016/j.cub.2010.11.012 21093266

[B19] Ingouff M. Selles B. Michaud C. Vu T. M. Berger F. Schorn A. J. (2017). Live-cell analysis of DNA methylation during sexual reproduction in *Arabidopsis* reveals context and sex-specific dynamics controlled by noncanonical RdDM. *Genes Dev.* 31 72–83. 10.1101/gad.289397.116 28115468 PMC5287115

[B20] Jacob Y. Bergamin E. Donoghue M. T. Mongeon V. LeBlanc C. Voigt P. (2014). Selective methylation of histone H3 variant H3.1 regulates heterochromatin replication. *Science* 343 1249–1253. 10.1126/science.1248357 24626927 PMC4049228

[B21] Jauvion V. Elmayan T. Vaucheret H. (2010). The conserved RNA trafficking proteins HPR1 and TEX1 are involved in the production of endogenous and exogenous small interfering RNA in *Arabidopsis*. *Plant Cell* 22 2697–2709. 10.1105/tpc.110.076638 20798330 PMC2947180

[B22] Li L. Wu W. Zhao Y. Zheng B. (2017). A reciprocal inhibition between ARID1 and MET1 in male and female gametes in *Arabidopsis*. *J. Integr. Plant Biol.* 59 657–668. 10.1111/jipb.12573 28782297

[B23] Lieber D. Lora J. Schrempp S. Lenhard M. Laux T. (2011). *Arabidopsis* WIH1 and WIH2 genes act in the transition from somatic to reproductive cell fate. *Curr. Biol.* 21 1009–1017. 10.1016/j.cub.2011.05.015 21658947

[B24] Lora J. Yang X. Tucker M. R. (2019). Establishing a framework for female germline initiation in the plant ovule. *J. Exp. Bot.* 70 2937–2949. 10.1093/jxb/erz212 31063548

[B25] Matzke M. A. Mosher R. A. (2014). RNA-directed DNA methylation: an epigenetic pathway of increasing complexity. *Nat. Rev. Genet.* 15 394–408. 10.1038/nrg3683 24805120

[B26] Mendes M. A. Petrella R. Cucinotta M. Vignati E. Gatti S. Pinto S. C. (2020). The RNA-dependent DNA methylation pathway is required to restrict SPOROCYTELESS/NOZZLE expression to specify a single female germ cell precursor in *Arabidopsis*. *Development* 147:194274. 10.1242/dev.194274 33158925 PMC7758631

[B27] Okada T. Endo M. Singh M. B. Bhalla P. L. (2005). Analysis of the histone H3 gene family in *Arabidopsis* and identification of the male-gamete-specific variant AtMGH3. *Plant J.* 44 557–568. 10.1111/j.1365-313X.2005.02554.x 16262706

[B28] Olmedo-Monfil V. Duran-Figueroa N. Arteaga-Vazquez M. Demesa-Arevalo E. Autran D. Grimanelli D. (2010). Control of female gamete formation by a small RNA pathway in *Arabidopsis*. *Nature* 464 628–632. 10.1038/nature08828 20208518 PMC4613780

[B29] Osipova T. N. Pospelov V. A. Svetlikova S. B. Vorob’ev V. I. (1980). The role of histone H1 in compaction of nucleosomes. sedimentation behaviour of oligonucleosomes in solution. *Eur. J. Biochem.* 113 183–188. 10.1111/j.1432-1033.1980.tb06153.x 7460945

[B30] Otero S. Desvoyes B. Peiro R. Gutierrez C. (2016). Histone H3 dynamics reveal domains with distinct proliferation potential in the *Arabidopsis* root. *Plant Cell* 28 1361–1371. 10.1105/tpc.15.01003 27207857 PMC4944401

[B31] Payne T. Johnson S. D. Koltunow A. M. (2004). KNUCKLES (KNU) encodes a C2H2 zinc-finger protein that regulates development of basal pattern elements of the *Arabidopsis* gynoecium. *Development* 131 3737–3749. 10.1242/dev.01216 15240552

[B32] Qin Y. Zhao L. Skaggs M. I. Andreuzza S. Tsukamoto T. Panoli A. (2014). ACTIN-RELATED PROTEIN6 regulates female meiosis by modulating meiotic gene expression in *Arabidopsis*. *Plant Cell* 26 1612–1628. 10.1105/tpc.113.120576 24737671 PMC4036575

[B33] Ravi M. Shibata F. Ramahi J. S. Nagaki K. Chen C. Murata M. (2011). Meiosis-specific loading of the centromere-specific histone CENH3 in *Arabidopsis thaliana*. *PLoS Genet* 7:e1002121. 10.1371/journal.pgen.1002121 21695238 PMC3111537

[B34] Ren L. Tang D. Zhao T. Zhang F. Liu C. Xue Z. (2018). OsSPL regulates meiotic fate acquisition in rice. *New Phytol.* 218 789–803. 10.1111/nph.15017 29479720

[B35] Rodriguez-Leal D. Leon-Martinez G. Abad-Vivero U. Vielle-Calzada J. P. (2015). Natural variation in epigenetic pathways affects the specification of female gamete precursors in *Arabidopsis*. *Plant Cell* 27 1034–1045. 10.1105/tpc.114.133009 25829442 PMC4558685

[B36] Rogers K. Chen X. (2013). Biogenesis, turnover, and mode of action of plant microRNAs. *Plant Cell* 25 2383–2399. 10.1105/tpc.113.113159 23881412 PMC3753372

[B37] Rojas-Gracia P. Roque E. Medina M. Rochina M. Hamza R. Angarita-Diaz M. P. (2017). The parthenocarpic hydra mutant reveals a new function for a SPOROCYTELESS-like gene in the control of fruit set in tomato. *New Phytol.* 214 1198–1212. 10.1111/nph.14433 28134991

[B38] Schiefthaler U. Balasubramanian S. Sieber P. Chevalier D. Wisman E. Schneitz K. (1999). Molecular analysis of NOZZLE, a gene involved in pattern formation and early sporogenesis during sex organ development in *Arabidopsis thaliana*. *Proc. Natl. Acad. Sci. U.S.A.* 96 11664–11669. 10.1073/pnas.96.20.11664 10500234 PMC18091

[B39] Schneitz K. Hulskamp M. Kopczak S. D. Pruitt R. E. (1997). Dissection of sexual organ ontogenesis: a genetic analysis of ovule development in *Arabidopsis thaliana*. *Development* 124 1367–1376. 10.1242/dev.124.7.1367 9118807

[B40] She W. Grimanelli D. Rutowicz K. Whitehead M. W. Puzio M. Kotlinski M. (2013). Chromatin reprogramming during the somatic-to-reproductive cell fate transition in plants. *Development* 140 4008–4019. 10.1242/dev.095034 24004947

[B41] Singh M. Goel S. Meeley R. B. Dantec C. Parrinello H. Michaud C. (2011). Production of viable gametes without meiosis in maize deficient for an ARGONAUTE protein. *Plant Cell* 23 443–458. 10.1105/tpc.110.079020 21325139 PMC3077773

[B42] Singh S. K. Kumar V. Srinivasan R. Ahuja P. S. Bhat S. R. Sreenivasulu Y. (2017). The TRAF mediated gametogenesis progression (TRAMGaP) gene is required for megaspore mother cell specification and gametophyte development. *Plant Physiol.* 175 1220–1237. 10.1104/pp.17.00275 28939625 PMC5664457

[B43] Su Z. Wang N. Hou Z. Li B. Li D. Liu Y. (2020). Regulation of female germline specification via small RNA mobility in *Arabidopsis*. *Plant Cell* 32 2842–2854. 10.1105/tpc.20.00126 32703817 PMC7474286

[B44] Su Z. Zhao L. Zhao Y. Li S. Won S. Cai H. (2017). The THO complex non-cell-autonomously represses female germline specification through the TAS3-ARF3 module. *Curr Biol* 27 1597–1609.e1592. 10.1016/j.cub.2017.05.021 28552357 PMC5544534

[B45] Talbert P. B. Masuelli R. Tyagi A. P. Comai L. Henikoff S. (2002). Centromeric localization and adaptive evolution of an Arabidopsis histone H3 variant. *Plant Cell* 14 1053–1066. 10.1105/tpc.010425 12034896 PMC150606

[B46] van Zanten M. Koini M. A. Geyer R. Liu Y. Brambilla V. Bartels D. (2011). Seed maturation in *Arabidopsis thaliana* is characterized by nuclear size reduction and increased chromatin condensation. *Proc. Natl. Acad. Sci. U.S.A.* 108 20219–20224. 10.1073/pnas.1117726108 22123962 PMC3250172

[B47] Wang H. Dittmer T. A. Richards E. J. (2013). *Arabidopsis* CROWDED NUCLEI (CRWN) proteins are required for nuclear size control and heterochromatin organization. *BMC Plant Biol.* 13:200. 10.1186/1471-2229-13-200 24308514 PMC3922879

[B48] Wei B. Zhang J. Pang C. Yu H. Guo D. Jiang H. (2015). The molecular mechanism of sporocyteless/nozzle in controlling *Arabidopsis* ovule development. *Cell Res.* 25 121–134. 10.1038/cr.2014.145 25378179 PMC4650584

[B49] Wu W. Li L. Zhao Y. Zhao Y. Jiang T. McCormick S. (2021). Heterochromatic silencing is reinforced by ARID1-mediated small RNA movement in *Arabidopsis* pollen. *New Phytol.* 229 3269–3280. 10.1111/nph.16871 32783185

[B50] Xiao W. Gehring M. Choi Y. Margossian L. Pu H. Harada J. J. (2003). Imprinting of the MEA polycomb gene is controlled by antagonism between MET1 methyltransferase and DME glycosylase. *Dev. Cell* 5 891–901. 10.1016/s1534-5807(03)00361-7 14667411

[B51] Yang W. C. Ye D. Xu J. Sundaresan V. (1999). The SPOROCYTELESS gene of *Arabidopsis* is required for initiation of sporogenesis and encodes a novel nuclear protein. *Genes Dev.* 13 2108–2117. 10.1101/gad.13.16.2108 10465788 PMC316961

[B52] Yao X. Yang H. Zhu Y. Xue J. Wang T. Song T. (2018). The canonical E2Fs are required for germline development in *Arabidopsis*. *Front. Plant Sci.* 9:638. 10.3389/fpls.2018.00638 29868091 PMC5962754

[B53] Yelina N. E. Smith L. M. Jones A. M. Patel K. Kelly K. A. Baulcombe D. C. (2010). Putative *Arabidopsis* THO/TREX mRNA export complex is involved in transgene and endogenous siRNA biosynthesis. *Proc. Natl. Acad. Sci. U.S.A.* 107 13948–13953. 10.1073/pnas.0911341107 20634427 PMC2922225

[B54] Zemach A. Kim M. Y. Hsieh P. H. Coleman-Derr D. Eshed-Williams L. Thao K. (2013). The *Arabidopsis* nucleosome remodeler DDM1 allows DNA methyltransferases to access H1-containing heterochromatin. *Cell* 153 193–205. 10.1016/j.cell.2013.02.033 23540698 PMC4035305

[B55] Zhao H. Guo M. Yan M. Cheng H. Liu Y. She Z. (2020). Comparative expression profiling reveals genes involved in megasporogenesis. *Plant Physiol.* 182 2006–2024. 10.1104/pp.19.01254 32054780 PMC7140934

[B56] Zhao L. Cai H. Su Z. Wang L. Huang X. Zhang M. (2018). KLU suppresses megasporocyte cell fate through SWR1-mediated activation of WRKY28 expression in *Arabidopsis*. *Proc. Natl. Acad. Sci. U.S.A.* 115 E526–E535. 10.1073/pnas.1716054115 29288215 PMC5776990

[B57] Zhao L. Liu L. Liu Y. Dou X. Cai H. Aslam M. (2021). Characterization of germline development and identification of genes associated with germline specification in pineapple. *Hortic Res.* 8:239. 10.1038/s41438-021-00669-x 34719672 PMC8558326

[B58] Zhao X. Bramsiepe J. Van Durme M. Komaki S. Prusicki M. A. Maruyama D. (2017). RETINOBLASTOMA RELATED1 mediates germline entry in *Arabidopsis*. *Science* 356:aaf6532. 10.1126/science.aaf6532 28450583

[B59] Zheng B. He H. Zheng Y. Wu W. McCormick S. (2014). An ARID domain-containing protein within nuclear bodies is required for sperm cell formation in *Arabidopsis thaliana*. *PLoS Genet* 10:e1004421. 10.1371/journal.pgen.1004421 25057814 PMC4109846

